# Clinical value of fetal facial profile markers during the first trimester

**DOI:** 10.1186/s12884-022-05028-9

**Published:** 2022-10-02

**Authors:** Xiaofeng Zhou, Chunya Ji, Lingling Sun, Linliang Yin, Xuedong Deng, Qi Pan, Jun Zhang, Zhong Yang, Chenhan Zheng, Chen Ling, Liping Shi, Yanqing Wu

**Affiliations:** 1 Department of Ultrasound, Changshu Hospital Affiliated to Nanjing University of Chinese Medicine, No. 6 Huanghe Street, Changshu, 215500 Jiangsu China; 2 Center for Medical Ultrasound, The Affiliated Suzhou Hospital of Nanjing Medical University, Suzhou Municipal Hospital, Gusu School, Nanjing Medical University, No. 26 Daoqian Street, Suzhou, 215002 Jiangsu China

**Keywords:** First trimester, Facial profile, Markers, Abnormal fetuses

## Abstract

**Objectives:**

To study the correlations between facial profile markers and crown-lump length (CRL) in a Chinese population, and to evaluate the clinical value of these markers for abnormal fetuses during the first trimester (11 to 13^+6^ gestational weeks).

**Methods:**

The facial profile markers were as followings: inferior facial angle (IFA), maxilla-nasion-mandible (MNM) angle, facial maxillary angle (FMA), frontal space (FS) distance and profile line (PL) distance. These markers were measured in facial mid-sagittal section through ViewPoint 6 software. The diagnostic value of these markers for abnormal fetuses was assessed by receiver operating characteristic (ROC) curves.

**Results:**

According to the first-trimester scanning (FTS) and follow-up, 31 fetuses were enrolled in the abnormal group, including 14 cases of trisomy 21, 7 cases of trisomy 18, 10 cases with cleft lip and palate (CLP), and 1000 normal fetuses were selected. Among the normal fetuses, the IFA, FS distance and PL distance had negative correlations with CRL. The MNM angle and FMA had positive correlations with CRL. The mean IFA values for fetuses with trisomy 21 and trisomy 18 were 74.11° (standard deviation (SD) 7.48) and 69.88° (SD 7.08), respectively, which were significantly smaller than the normal fetuses (*p* = 0.013; *p* = 0.003). The mean MNM angle of fetuses with trisomy 18 and CLP were 6.98° (SD 2.61) and 9.41° (SD 2.57), respectively, which were significantly greater than the normal fetuses (*p* = 0.005; *p* < 0.001). The mean FMA values of trisomy 18 fetuses were 63.95° (SD 4.77), which was significantly smaller than the normal fetuses (*p* < 0.001). The mean FS distance of CLP fetuses was -0.22 mm (SD 1.38), which was significantly smaller than the normal fetuses (*p* < 0.001). The mean PL distance of trisomy 21, trisomy 18 and CLP fetuses were 2.89 mm (SD 0.41), 2.91 mm (SD 0.56) and 2.71 mm (SD 0.37), respectively. The difference with the normal fetuses had no statistical significance (*p* = 0.56; *p* = 0.607; *p* = 0.54).

**Conclusions:**

Fetal facial profile markers had excellent correlations with CRL during the first trimester. IFA had certain clinical significance in detecting trisomy 21. FMA, IFA and MNM angle were reliable indicators for screening trisomy 18. The abnormal MNM angle and FS distance could be used as sensitive indicators for CLP. However, PL distance was not the best markers for trisomy 21, trisomy 18 and CLP.

**Supplementary Information:**

The online version contains supplementary material available at 10.1186/s12884-022-05028-9.

## Background

Facial malformations are the most common fetal surface malformations, including cleft lip and palate (CLP), micrognathia and so on. It not only brings great adverse effect to the children and their family, but also aggravates the social burden [1, 2]. With the advanced development of nuchal translucency (NT) scan and chromosomal aneuploidy risk assessment during the first trimester, the screening time for fetal structural abnormalities has been advanced from the mid-trimester to the first trimester, which provides adequate time to pregnant women for genetic counseling and reasonable choice of pregnant outcome [3].

The fetal facial profile contains abundant information. The fetal facial mid-sagittal section is an important section to evaluate the facial profile in first-trimester scanning (FTS). In this section, not only the NT thickness can be measured, but also such facial structures as the forehead, nasal bone, palate, upper lip, lower lip and mandible can be observed from top to bottom. In recent years, some scholars have converted these complex facial structures into objective ultrasonographic measured markers, such as inferior facial angle (IFA), maxilla-nasion-mandible (MNM) angle, facial maxillary angle (FMA), frontal space (FS) distance and profile line (PL) distance, et al. Some studies have reported that the abnormal values of facial markers are closely related to fetal chromosomal abnormalities (such as trisomy 21, trisomy 18) and facial deformities (such as CLP, micrognathia) [4–10]. However, studies on these markers are mainly concentrated in the second and third trimester, and there are few reports during the first trimester. The purpose of this study was to study the correlations between facial profile markers and crown-lump length (CRL) in a Chinese population, and to evaluate the early diagnostic value of these markers for abnormal fetuses (such as trisomy 21, trisomy 18 and CLP) during the first trimester.

## Methods

### Study subjects

The normal fetuses had normal ultrasound and normal follow-up outcomes. Fetuses diagnosed as chromosomal abnormalities by invasive genetic diagnosis or those with facial structural abnormalities suspected by ultrasound and confirmed after induced abortion or birth were categorized as the abnormal group. Ultrasound images of FTS, performed in the Affiliated Suzhou Hospital of Nanjing Medical University during August 2017 to December 2020, were selected. We have included all the cases in the selected time frame without a priori calculation of the sample size.

Inclusion criteria: (1) both parents of the fetus were Chinese; (2) singleton pregnancy; (3) Two-dimensional (2D) stored images were the fetal facial mid-sagittal section, which strictly followed the guidelines of Fetal Medicine Foundation (FMF). The forehead, nasal bone, palate, mandible, upper lip and lower lip of the fetuses were clearly distinguishable; (4) There was no umbilical cord or limb in front of the fetal face.

Exclusion criteria: (1) pregnancies with significant maternal complications; (2) fetuses were lost to follow-up.

This study was approved by the Ethics Committee of the Affiliated Suzhou Hospital of Nanjing Medical University (K2016038). Pregnant women voluntarily participated in the follow-up and signed informed consent forms.

### Equipment and software

A GE Voluson E8 Expert and a Philips Affiniti70 four-dimensional (4D) color ultrasound diagnostic instruments were used in this study. The ultrasound examinations were performed transabdominally, using convex probe C5-1 (frequency 1 ~ 5 MHz) and C9-2 (frequency 2 ~ 9 MHz). The image was stored in DICOM (Digital Imaging and Communications in Medicine) format and the measurement software was ViewPoint 6 ultrasound workstation.

### Ultrasonic measurement of facial profile markers

**Fig. 1:**
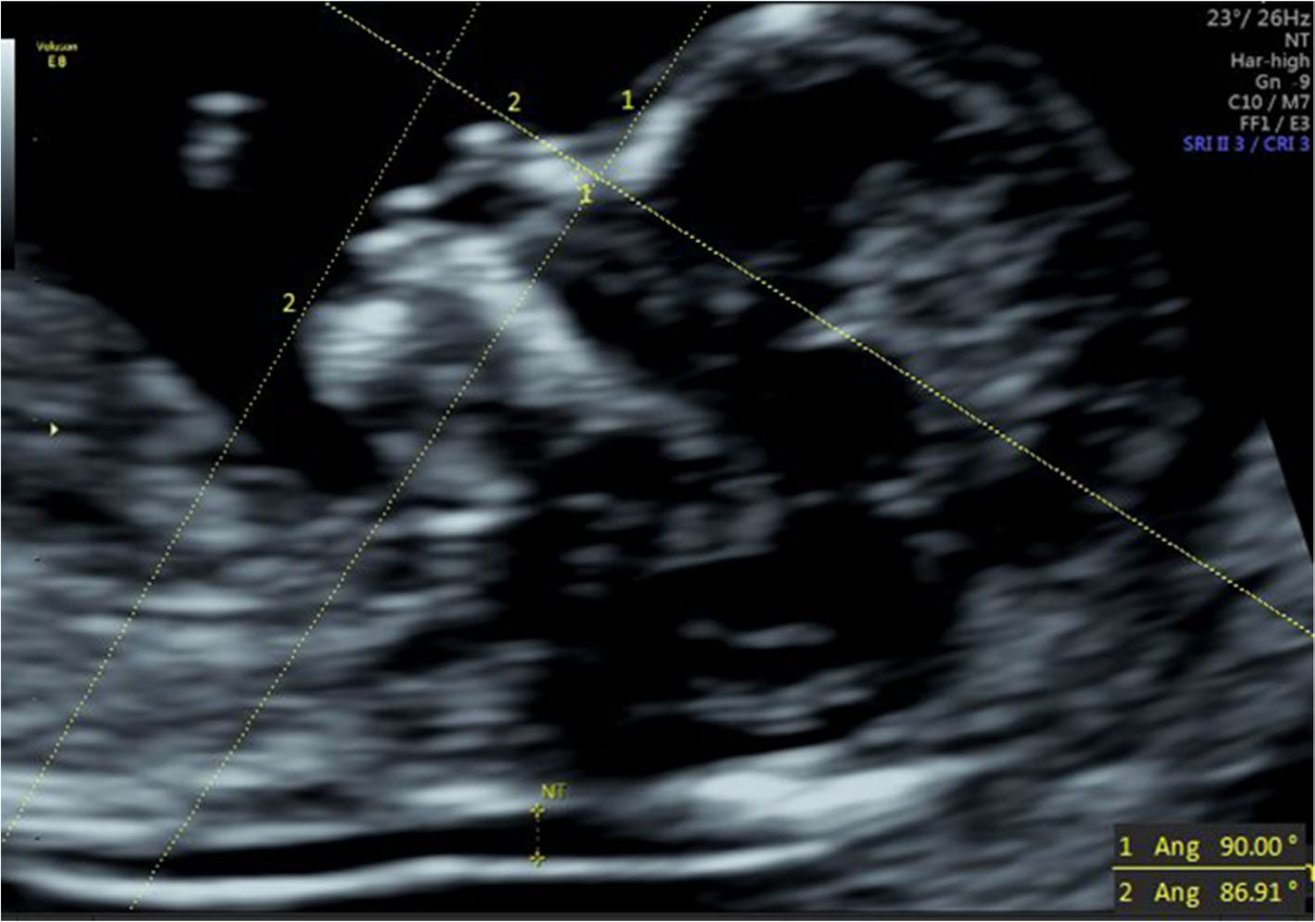
The measurement of IFA (86.91°); 12w6d, normal Chinese fetus

**Fig. 2:**
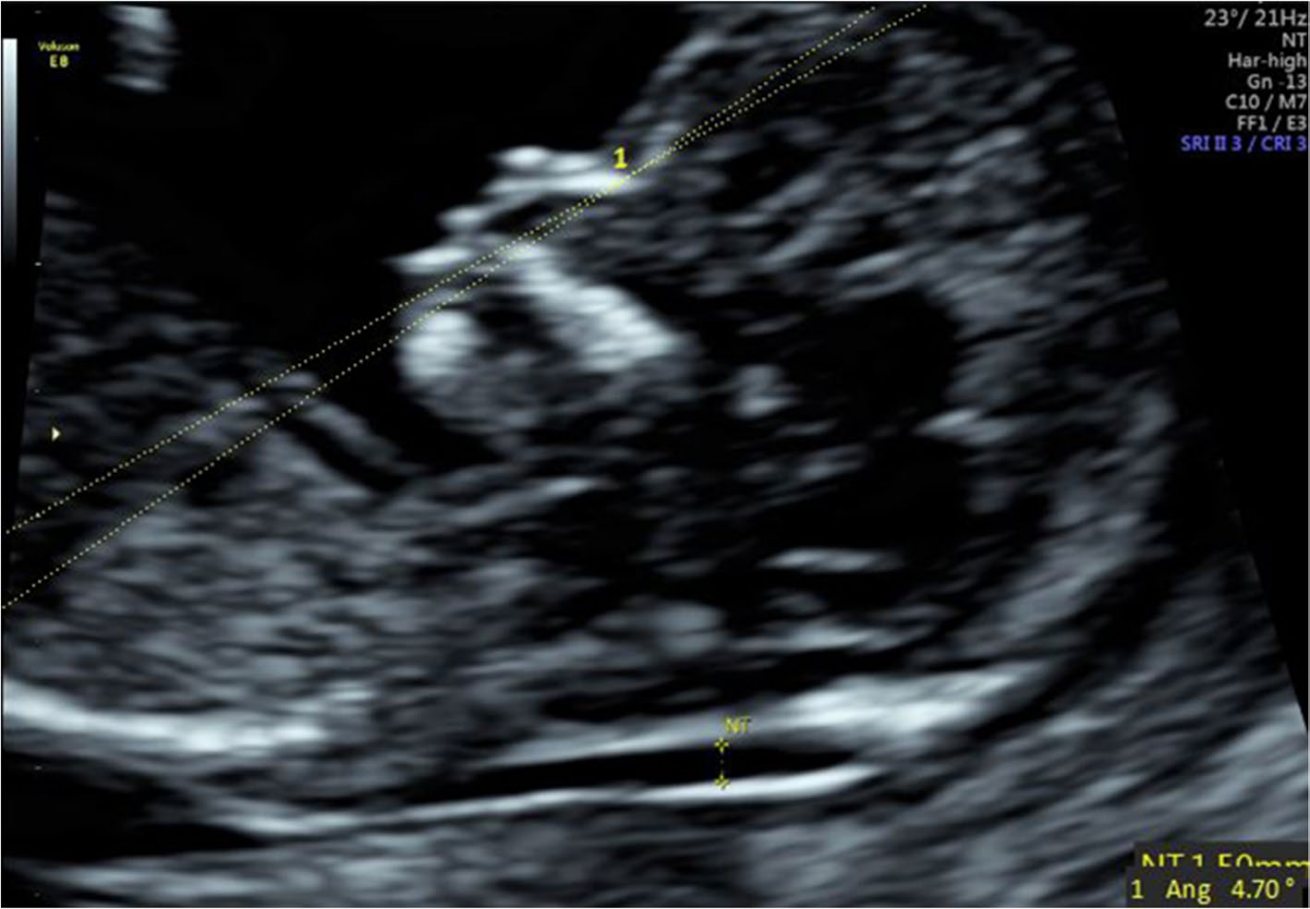
The measurement of MNM angle (4.7°); 13w1d, normal Chinese fetus

**Fig. 3:**
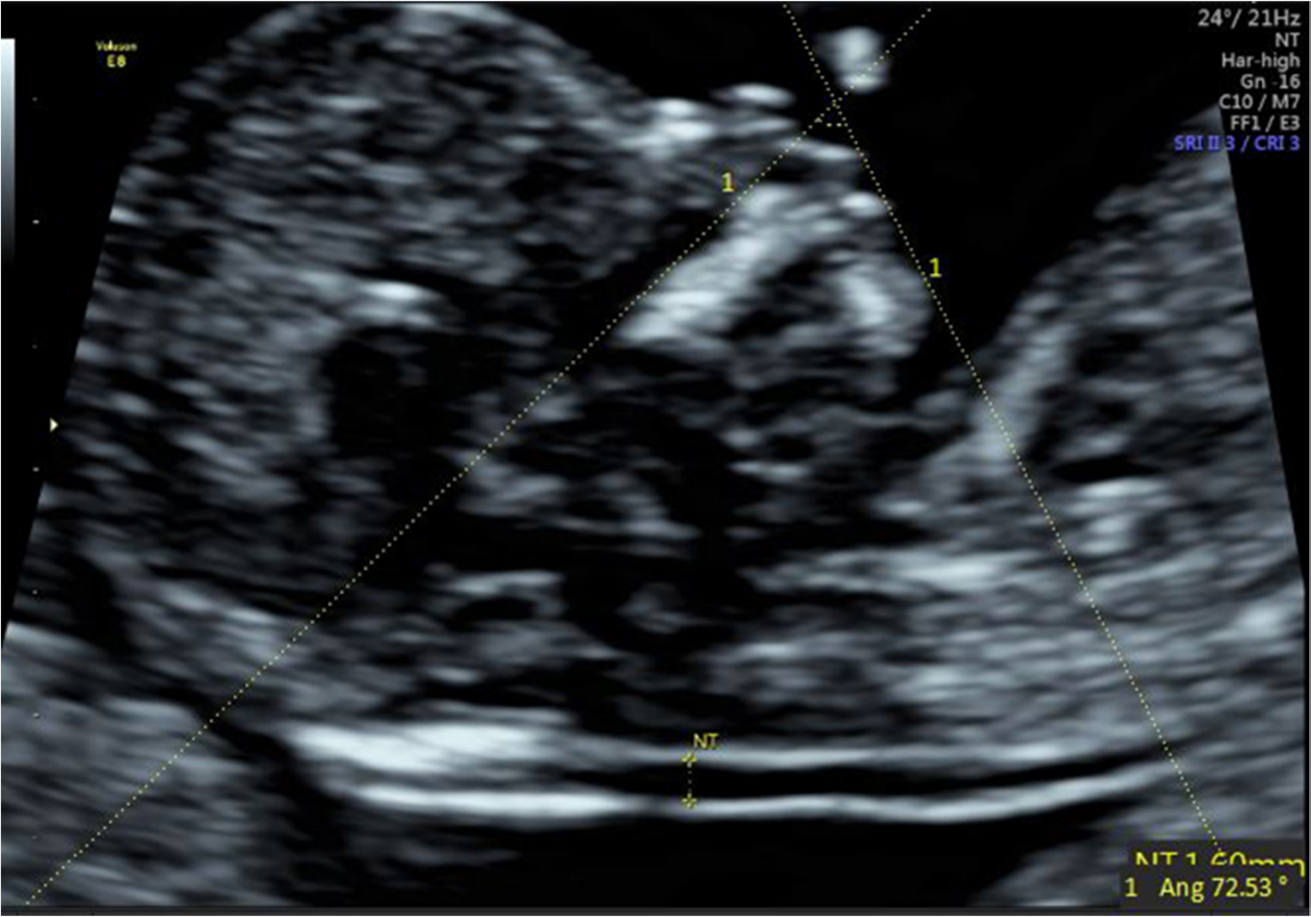
The measurement of FMA (72.53°); 13w3d, normal Chinese fetus

**Fig. 4:**
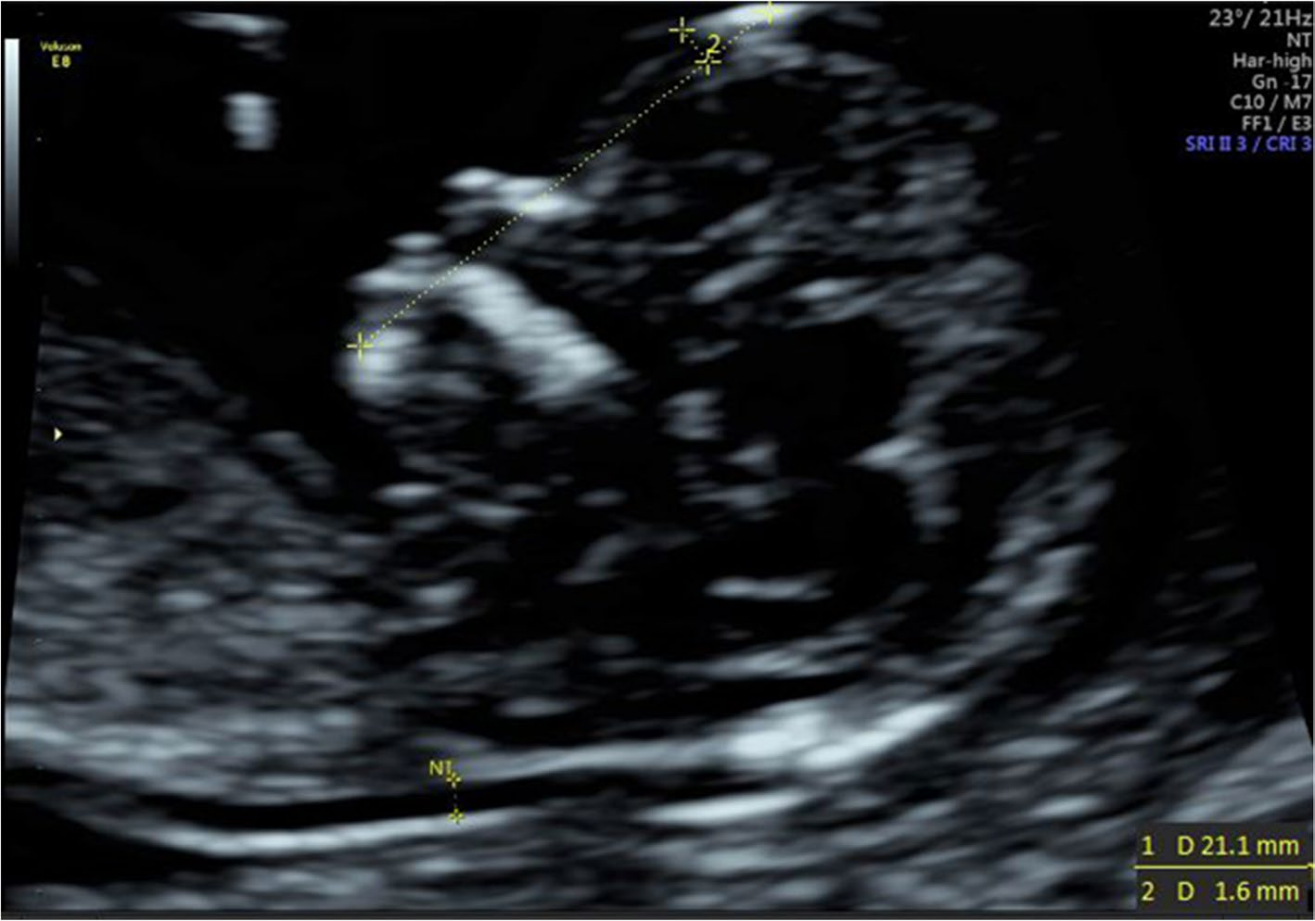
The measurement of FS distance (1.6 mm); 13w1d, normal Chinese fetus

**Fig. 5:**
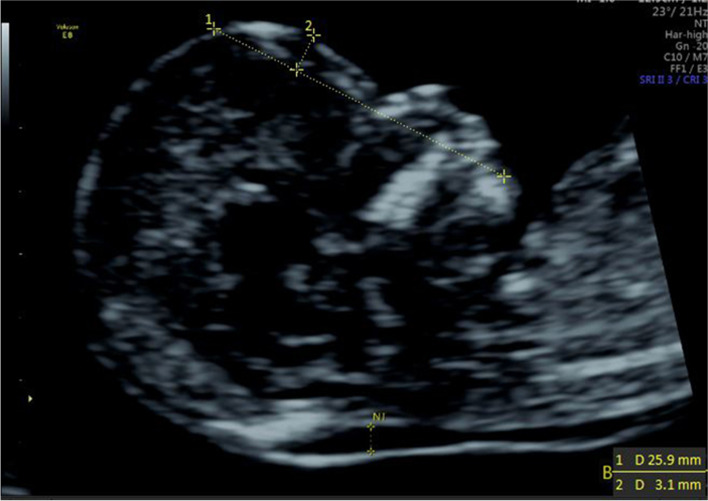
The measurement of PL distance (3.1 mm); 13w1d, normal Chinese fetus

- (1) IFA [4, 5, 11]: the angle between the line orthogonal to the vertical part of the forehead at the level of the synostosis of the nasal bones and a second line joining the tip of the mentum to the anterior point of the more protruding lip (Fig. 1).
- (2) MNM angle [6, 11]: the angle between the maxilla–nasion line and the mandible–nasion line (Fig. 2). The nasion [10, 11] was defined as the most anterior point at the intersection of the frontal and nasal bone.
- (3) FMA [7, 11]: the angle between the line overlying the maxilla and the line across mentum tip and upper lip (Fig. 3).
- (4) FS distance [8, 9]: the maximum perpendicular distance from the mandibulo-maxillary line (MML) to the most prominent part of the fetal forehead (Fig. 4). The MML [8] was an extended line intersecting the most anterior portions of the mandible and the maxilla.
- (5) PL distance [10, 11]: the maximum perpendicular distance from the facial profile line (FPL) to the outer border of the forehead (Fig. 5). The FPL [10, 11] was the line passing through the middle point of the anterior border of the mandible and the nasion.

These facial markers were measured through ViewPoint 6 software by two experienced sonographers, who had obtained the NT screening qualification certificate issued by the FMF.

Neither of the two surveyors knew the fetal karyotype, and the mean value of each marker was taken after three measurements. It took 5 to 6 min for three measurements of all 5 markers.

### Statistical analysis

All statistical analysis was performed by SPSS21.0 (Chicago, IL, USA) and Graphpad Prism8.0. Continuous variables with a Gaussian distribution were expressed as mean (standard deviation, SD). Continuous variables without a Gaussian distribution were expressed by Median (Inter-Quartile Range, IQR). Spearman’s rank correlation test was used to analyze the correlations between fetal facial markers and CRL. The Student’s T test or Mann–whitney U test were used to analyze the differences among the groups. When *p* < 0.05, the differences were considered as statistically significant. The diagnostic value of these markers for abnormal fetuses was assessed by receiver operating characteristic (ROC) curves.

## Results

### Data of the normal fetuses and correlations between fetal facial markers and CRL

One thousand normal fetuses whose image was clear and typical for measurement were selected. The median maternal age was 28 years (IQR 26–31). The median CRL was 66 mm (IQR 62–71), and the median NT thickness was 1.6 mm (IQR 1.3–1.9).

IFA was distributed from 55.9° to 107.89°, and the median was 78.25° (IQR 74.75–82.98). Spearman’s rank correlation analysis showed that IFA was negatively correlated with CRL during the first trimester (r_s_ = -0.614, *p* < 0.001). MNM angle was distributed from 1.38° to 10.7°, and the median was 4.07° (IQR 3.21–5.29). MNM angle depended significantly on CRL during the first trimester (r_s_ = 0.574, *p* < 0.001). FMA was distributed from 56.29° to 91.87°, and the mean was 73.02° (SD 5.01). FMA had significant positive correlation to CRL (r_s_ = 0.451, *p* < 0.001). FS distance was distributed from 0 to 4.3 mm, and the median was 1.97 mm (IQR 1.53–2.37). FS distance decreased with CRL (r_s_ = -0.42, *p* < 0.001). PL distance was distributed from 1.1 to 4.67 mm, and the mean was 2.81 mm (SD 0.53). PL distance decreased with CRL (r_s_ = -0.271, *p* < 0.001).

### Comparison of facial profile markers between the abnormal fetuses and normal fetuses

**Fig. 6:**
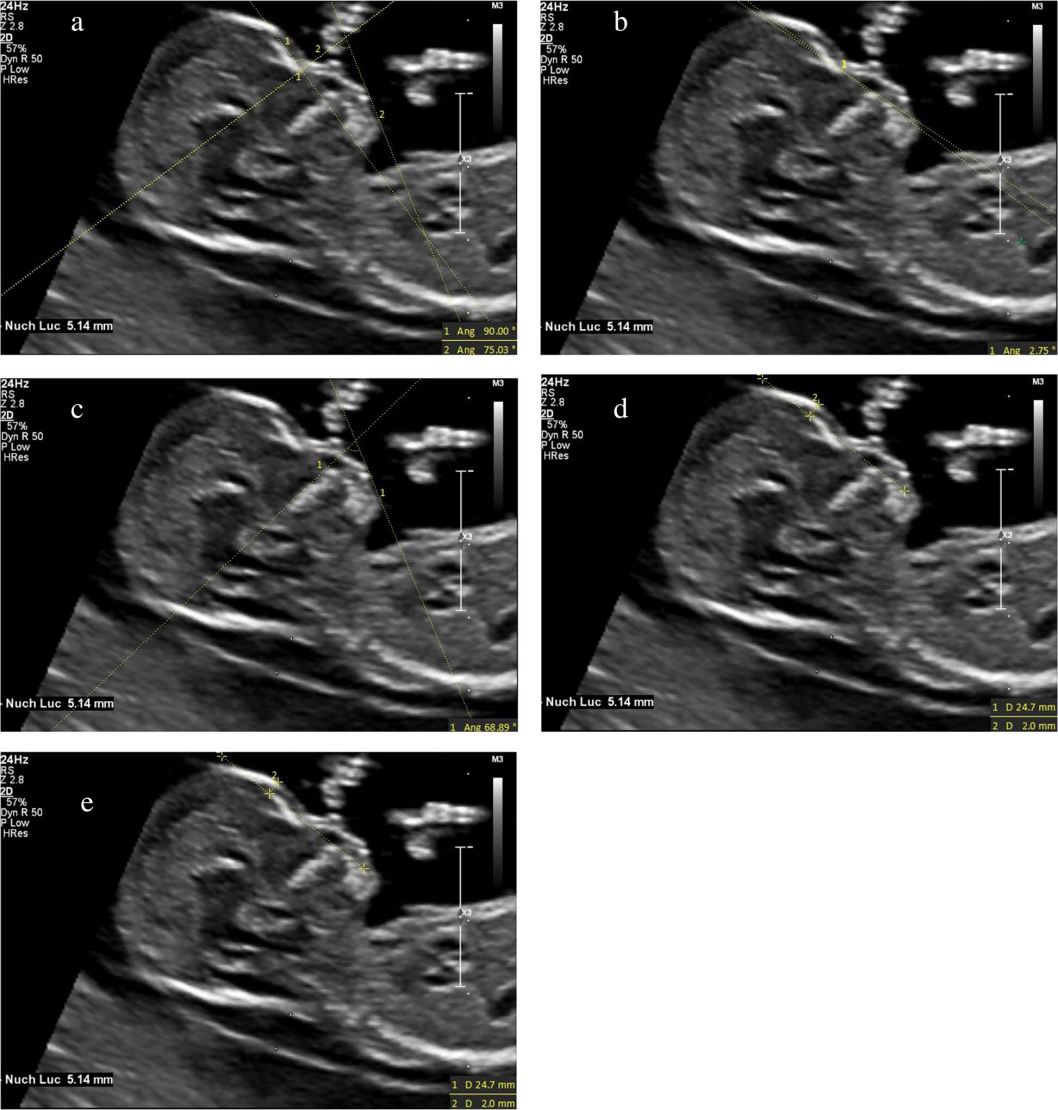
The measurements of each facial marker of trisomy 21 fetus; 12w5d. **a** IFA 75.03°; **b** MNM angle 2.75°; **c** FMA 68.89°; **d** FS distance 2.0 mm; **e** PL distance 2.5 mm

**Fig. 7:**
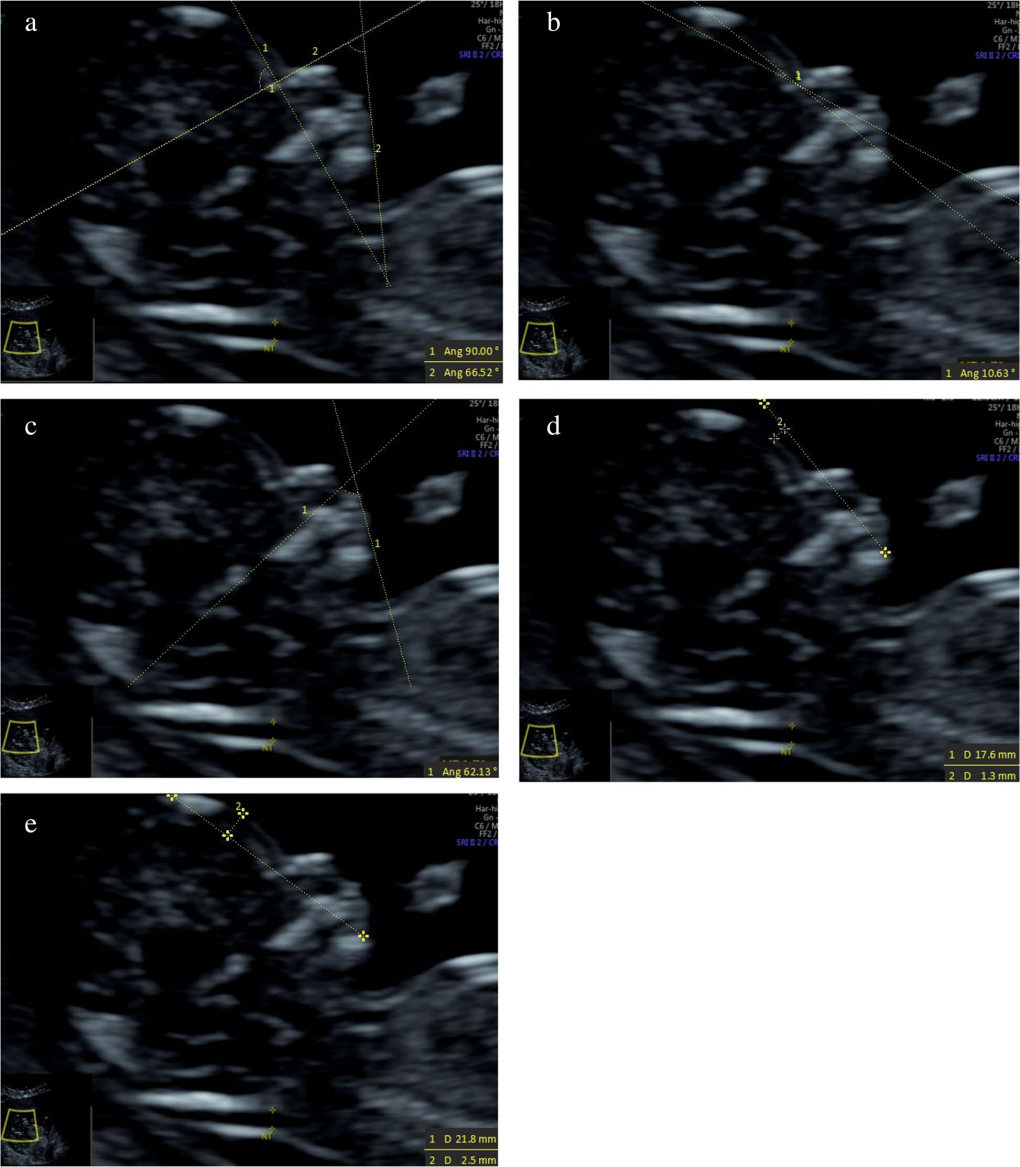
The measurements of each facial marker of trisomy 18 fetus; 12w4d. **a** IFA 66.52°; **b** MNM angle 10.63°; **c** FMA 62.13°; **d** FS distance 1.3 mm; **e** PL distance 2.5 mm

**Fig. 8:**
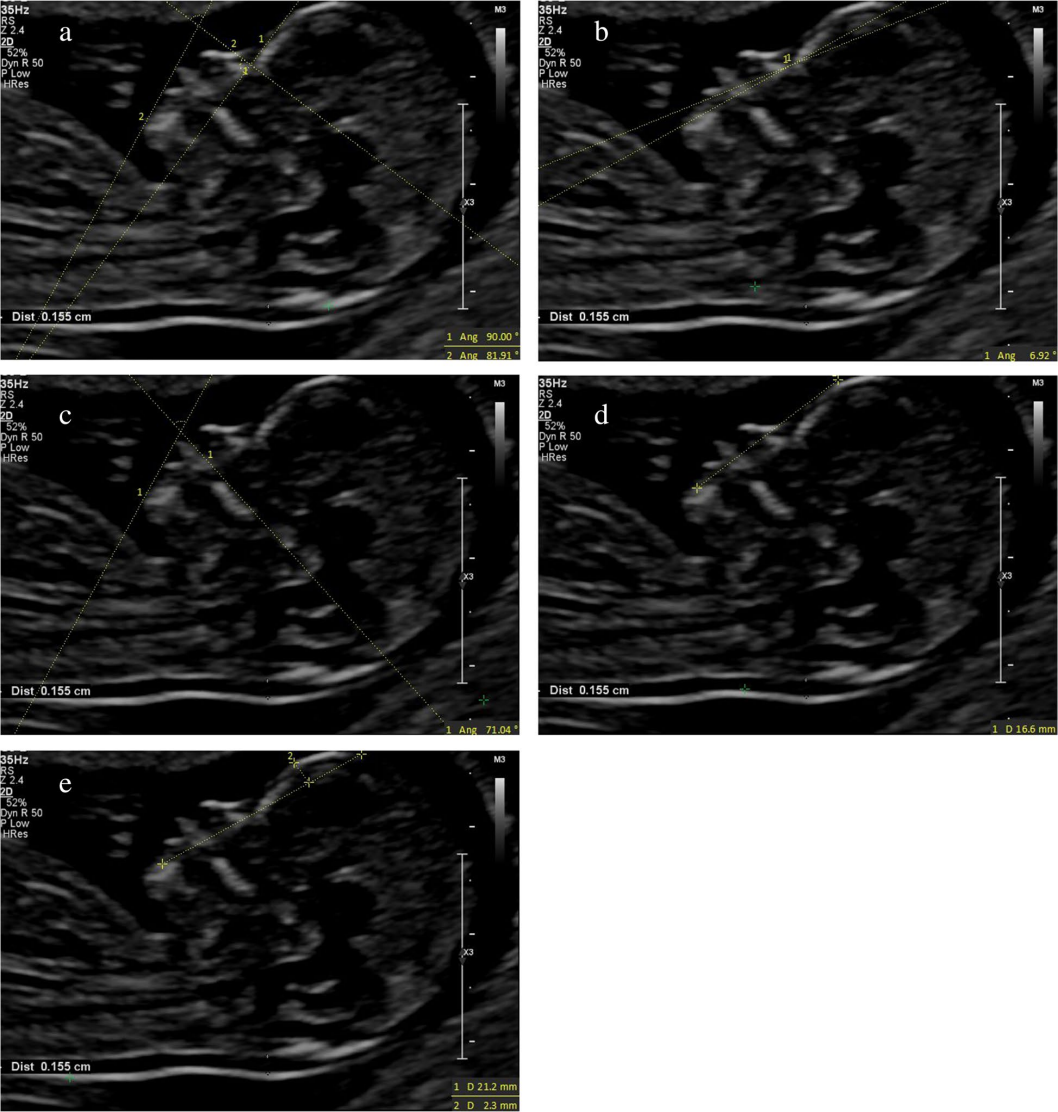
The measurements of each facial marker of CLP fetus; 13w1d. **a** IFA 81.91°; **b** MNM angle 6.92°; **c** FMA 71.04°; **d** FS distance 0 mm; **e** PL distance 2.3 mm

Thirty-one fetuses were selected in the abnormal group, including 14 cases of trisomy 21, 7 cases of trisomy 18, 10 cases of CLP. The mean maternal age was 32.48 years (SD 5.32). The mean CRL was 65.65 mm (SD 6.23), and the mean NT thickness was 3.13 mm (SD 1.84). Ultrasonographic measurements of each facial marker of trisomy 21, trisomy 18 and CLP fetuses were shown in Figs. 6a-e, 7a-e and 8a-e, respectively. Ultrasonographic measurement results of fetal facial markers in the normal fetuses and abnormal group were shown in Table 1.

**Table 1:** The mean (SD)/median (IQR) value for IFA, MNM angle, FMA, FS distance and PL distance in the normal fetuses and abnormal group, respectively

	Normal	Abnormal		
		Trisomy 21	Trisomy 18	CLP
IFA	78.25° (74.75–82.98)	74.11° (7.48)	69.88° (7.08)	82.03° (11.52)
MNM angle	4.07° (3.21–5.29)	4.59° (1.71)	6.98° (2.61)	9.41° (2.57)
FMA	73.02° (5.01)	74.33° (6.25)	63.95° (4.77)	74.42° (7.62)
FS distance	1.97 mm (1.53–2.37)	2.26 mm (0.85)	1.70 mm (1.81)	-0.22 mm (1.38)
PL distance	2.81 mm (0.53)	2.89 mm (0.41)	2.91 mm (0.56)	2.71 mm (0.37)
Cases	1000	14	7	10

*IFA* inferior facial angle, *MNM angle* maxilla-nasion-mandible angle, *FMA* facial maxillary angle, *FS distance* frontal space distance, *PL distance* profile line distance

The mean IFA values of trisomy 21 and trisomy 18 fetuses were 74.11° (SD 7.48) and 69.88° (SD 7.08), respectively, which were significantly smaller than the normal fetuses (Z = -2.496, *p* = 0.013; Z = -3.018, *p* = 0.003). The mean IFA values of CLP fetuses were 82.03° (SD 11.52). The difference with the normal fetuses had no statistical significance (Z = -1.495, *p* = 0.135).

The mean MNM angle of trisomy 18 and CLP fetuses were 6.98° (SD 2.61) and 9.41° (SD 2.57), respectively, which was significantly greater than the normal fetuses (Z = -2.83, *p* = 0.005; Z = -5.05, *p* < 0.001). The mean MNM angle of trisomy 21 were 4.59° (SD 1.71). The difference with the normal fetuses had no statistical significance (Z = -0.386, *p* = 0.7).

The mean FMA values of trisomy 18 fetuses were 63.95° (SD 4.77), which was significantly smaller than the normal fetuses (t = 4.777, *p* < 0.001). The mean FMA values of trisomy 21 and CLP were 74.33° (SD 6.25) and 74.42° (SD 7.62), respectively, which had no statistically significant difference (t = -0.967, *p* = 0.334; t = -0.577, *p* = 0.578).

The mean FS distance of CLP fetuses was -0.22 mm (SD 1.38), which was significantly smaller than the normal fetuses (Z = -4.259, *p* < 0.001). The mean FS distance of trisomy 21 and trisomy 18 fetuses were 2.26 mm (SD 0.85) and 1.70 mm (SD 1.81), respectively, which had no statistically significant difference (Z = -1.23, *p* = 0.219; Z = 0.277, *p* = 0.782).

The mean PL distance of trisomy 21, trisomy 18 and CLP fetuses were 2.89 mm (SD 0.41), 2.91 mm (SD 0.56) and 2.71 mm (SD 0.37), respectively. The difference with the normal fetuses had no statistical significance (t = -0.583, *p* = 0.56; t = -0.515, *p* = 0.607; t = 0.613, *p* = 0.54).

**Fig. 9:**
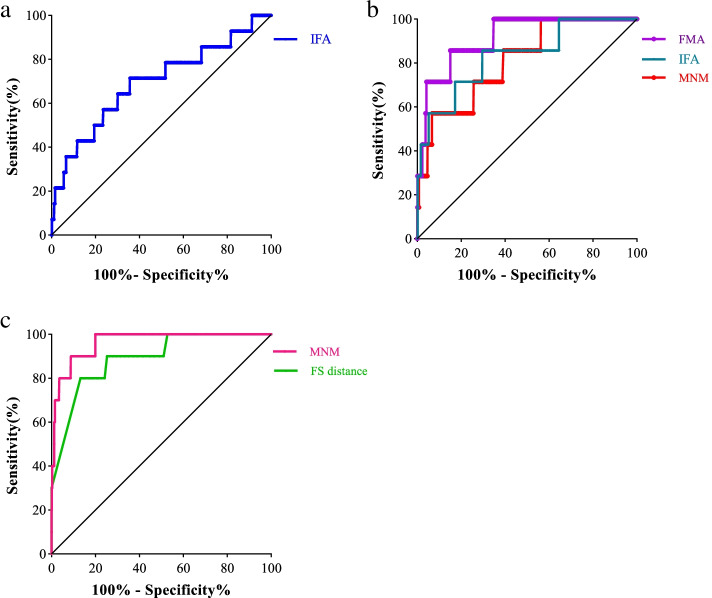
ROC curve of IFA for trisomy 21 fetuses (**a**); IFA, MNM angle and FMA for trisomy 18 fetuses (**b**); MNM angle and FS distance for CLP fetuses (**c**)

ROC curve was used to evaluate the diagnostic value of IFA for trisomy 21 fetuses; IFA, MNM angle and FMA for trisomy 18 fetuses; MNM angle and FS distance for CLP fetuses, respectively, as shown in Fig. 9a-c. The area under curve (AUC) of each ROC curve and its corresponding *p* value were shown in Table 2.

**Table 2:** The diagnostic value and cut-off of IFA for trisomy 21 fetuses; IFA, MNM angle and FMA for trisomy 18 fetuses; MNM angle and FS distance for CLP fetuses

	Trimosy 21	Trimosy 18			CLP	
	IFA	IFA	MNM angle	FMA	MNM angle	FS distance
AUC	0.694	0.831	0.81	0.914	0.964	0.89
95% CI	0.536 ~ 0.852	0.668 ~ 0.993	0.659 ~ 0.961	0.827 ~ 1	0.926 ~ 1	0.794 ~ 0.987
Sensitivity	0.643	0.704	0.933	0.85	0.912	0.868
Specificity	0.714	0.857	0.571	0.857	0.9	0.8
Cut-off	76.34°	75.37°	7.24°	67.96°	6.83°	0.35 mm
*p* value	0.013^a^	0.003^a^	0.005^a^	*p* < 0.001^a^	*p* < 0.001^a^	*p* < 0.001^a^

*AUC* area under curve, *CI* confidence interval, *CLP* cleft lip and palate, *IFA* inferior facial angle, *MNM angle* maxilla-nasion-mandible angle, *FMA* facial maxillary angle, *FS distance* frontal space distance

^a^Denotes significant values if *p* < 0.05

## Discussion

Facial profile markers during the first trimester is composed of facial bones. The development of facial bones is related to the migration and differentiation of neural crest cells from ectoderm, and presents a certain sequence [12]. At the 4th week of embryonic development, there are five protuberances around the primitive oral cavity: the frontonasal protuberance, the paired mandibular protuberances and the maxillary protuberances [13]. CLP is due to the growth and fusion disorders of these five protuberances [14]. The ossification of maxilla and mandible begins from 8 weeks onward [15]. During the first trimester, the mandible is indirectly connected to the skull through the temporomandibular joint and muscles, and the forward growth rate is not as fast as that of the maxilla, which is directly connected to the skull [16, 17]. From 20 weeks of gestation, the development of fetal swallowing function accelerates the growth of the mandible, while the maxilla ossification has initially completed. Subsequently, the position of the facial bones is relatively constant, and the fetal facial profile is basically formed [17]. In this study, multiple facial markers reflecting the relative position of forehead, maxilla, nasion and mandible were selected to analyze their early diagnostic value for trisomy 21, trisomy 18 and CLP fetuses during the first trimester.

In 2002, Rotten et al. [4] first introduced a quantitative facial angle called IFA to describe the relative position between mandible and frontal bone. They found that IFA was below the 5th percentile in 25% of trisomy 21 fetuses during the second and third trimester. When it was below 49.2° (average-2SD), micrognathia could be suspected, with a sensitivity of 100%, a specificity of 98.9%, a positive predictive value of 75% and a negative predictive value of 100%. IFA was mainly used to objectively evaluate the micrognathia, so as to indicate Pierre-Robin syndrome, Stickler syndrome, trisomy 18 and trisomy 13 [5, 18, 19]. In our study, the IFA values of trisomy 21 and trisomy 18 fetuses were significantly smaller than the normal fetuses. Moreover, the diagnostic accuracy of trisomy 18 was much higher than trisomy 21 (83.1% > 69.4%). IFA below 75.37° during the first trimester was significant for suggesting trisomy 18, with a sensitivity of 70.4% and specificity of 85.7%. This cut-off was much larger than Rotten et al. [4] (49.2°), which might be due to the slower forward growth rate of mandible than maxilla during the first trimester. However, the clinical significance of the cut-off need to be confirmed by a large sample study.

The MNM angle could reflect the relative position of maxilla, nasion and mandible. Bakker et al. [10] reported the MNM angle abnormally increased (above the 97.5th percentile) in bilateral CLP and micrognathia during the first trimester, confirming the predictive value in abnormal facial profile and special facial malformations. According to Lu et al. [7], in 4 cases of trisomy 18 with micrognathia, the MNM angle values were above the upper limit of normal values, which were similar to the results of de Jong-pleij et al. [6]. Similarly, in our study, the MNM angle was above the 95th percentile of normal values in 42.9% of trisomy 18 and 80% of CLP fetuses. However, it had an accuracy of 96.4%, a sensitivity of 91.2% and a specificity of 90% to detect CLP, which was superior to detecting trisomy 18. Therefore, we boldly speculated that the MNM angle was a sensitive indicator for judging facial profile during the first trimester. When it abnormally increased and was above 6.83°, the possibility of CLP should be considered. Meanwhile, we found that there was no statistically significant difference in MNM angle between trisomy 21 and the normal fetuses. It might be related to the fact that most trisomy 21 fetuses were accompanied by midfacial hypoplasia, low tongue tension and outside oral cavity [20]. Another possible explanation was that trisomy 21 had hypoplasia of the maxilla as well as hypoplasia of the mandible to a less degree [21, 22]. In that case, the position of the maxilla and mandible was changed, but the angle between them (MNM angle) might remain normal.

FMA could directly reflect the relative position of maxilla and mandible.

Lu et al. [7] observed that the variation of the value of FMA changed very little after 16 weeks’ gestation (the minimum was 64.6° at 16 weeks, and the maximum was 67.1° at 28 weeks). Using 66° as a cut-off, FMA had a detection rate of 100% for micrognathia (the false positive rate as low as 2.5%). In our study, the FMA values of trisomy 18 were significantly smaller than the normal fetuses. The cut-off of FMA in diagnosing trisomy 18 was 67.96°, with a sensitivity of 85% and a specificity of 85.7%. The value was similar to the results of Lu et al. [7]. A large prospective cohort was needed to confirm the accuracy of FMA in the diagnosis of trisomy 18 during the first trimester.

The FS distance could be used to evaluate the relative position of maxilla, mandible and frontal bone on the basis of MML. Yazdi et al. [8] showed that when FS distance was added into the first-trimester combined screening for aneuploidy, the false positive rate could be reduced from 5 to 3% while the detection rate of aneuploidy (90%) remained unchanged. In 2016, Hoopmann et al. [9] pointed out abnormal FS distance could detect some tiny maxillary protrusion, which had a definite implication for fetal CLP during the first trimester. Similarly, we found that the FS distance of CLP was significantly smaller than the normal fetuses. Setting the cut-off to 0.35 mm, the FS distance had an accuracy of 89%, a sensitivity of 86.8% and a specificity of 80% to diagnose CLP. Once abnormal FS distance was found, it was necessary to pay attention to whether the fetus had facial malformations (such as CLP) and chromosomal abnormalities.

The PL distance was affected by the position of mandible, nasion and frontal bone. Jong-pleij et al. [23] pointed out that the PL distance above 4 mm was an objective indicator to predict frontal bossing during the second and third trimester. Nevertheless, in our study we concluded that the PL distance of trisomy 21, trisomy 18 and CLP fetuses had no significant difference from the normal fetuses. Therefore, PL distance was not the best ultrasound markers for trisomy 21, trisomy 18 and CLP during first trimester. This is consistent with Bakker et al. [10]. However, we need a large number of prospective studies to verify it.

The main limitation of our study is the number of abnormal cases. It’s a bit less. A large multi centered prospective cohort study is required. Secondly, 6 cases of trisomy 21 and 1 case of trisomy 18 fetuses had absent or shortened nasal bone, and the other 14 cases of trisomy fetuses showed no obvious facial malformations. Thirdly, chromosomal analysis was not performed in 10 cases of CLP fetuses due to personal reasons, which limited the analysis of results to a extent. Additionally, 2D ultrasound images were used in this study. Some scholars considered that three-dimensional (3D) ultrasound could obtain a real standard mid-sagittal section through multi-plane mode [24]. However, numerous researches show that 2D ultrasound is the basis of 3D ultrasound reconstruction imaging and 10% of 3D reconstructed images could not be used for NT evaluation [25]. As a consequence, the measurement of facial markers by 2D ultrasound was easier for clinical application. Lastly, it was worth mentioning that the measurement of these facial markers was time-consuming and complicated. In the future, we will integrate artificial intelligence (AI) with these markers to promote the establishment of intelligent medical mode and increase the efficiency.

## Conclusion

There was a good correlation between fetal facial profile markers and CRL during the first trimester in a Chinese population. IFA had a certain value in the diagnosis for trisomy 21 and trisomy 18, with the cut-off of 76.34° and 75.37°, respectively. The advantage of MNM angle in detecting CLP was superior to trisomy 18. When the MNM angle was above 6.83° or FS distance below 0.35 mm, the possibility of CLP should be considered. Among FMA, IFA and MNM angle, FMA had the highest accuracy (91.4%) in detecting trisomy 18, with a cut-off of 67.96°. These cut-off need to be confirmed by a large number of prospective studies. While PL distance was not the best ultrasound markers for trisomy 21, trisomy 18 and CLP during first trimester.

## Supplementary Information

**Additional file 1.** 

## Data Availability

The datasets and code are not publicly available due to the hospital policy and personal privacy, but are available from the corresponding author on reasonable request.

## Abbreviations

AI: Artificial intelligence
AUC: Area under curve
CI: Confidence interval
CLP: Cleft lip and palate
CRL: Crown-rump length
DICOM: Digital Imaging and Communications in Medicine
FMA: Facial maxillary angle
FMF: Fetal Medicine Foundation
FPL: Facial profile line
FS distance: Frontal space distance
FTS: First-trimester scanning
IFA: Inferior facial angle
IQR: Inter-Quartile Range
MML: Mandibulo-maxillary line
MNM: Maxilla-nasion-mandible angle
NT: Nuchal translucency
PL distance: Profile line distance
ROC: Receiver operating characteristic
SD: Standard deviation
2D-US: Two-dimensional ultrasound
4D: Four-dimensional
